# The prognostic relevance of primary tumor location in patients undergoing resection for pancreatic ductal adenocarcinoma

**DOI:** 10.18632/oncotarget.14768

**Published:** 2017-01-20

**Authors:** Qi Ling, Xiao Xu, Panpan Ye, Haiyang Xie, Feng Gao, Qichao Hu, Zhikun Liu, Xuyong Wei, Christian Röder, Anna Trauzold, Holger Kalthoff, Shusen Zheng

**Affiliations:** ^1^ Department of Surgery, Collaborative Innovation Center for Diagnosis and Treatment of Infectious Diseases, The First Affiliated Hospital, College of Medicine, Zhejiang University, Hangzhou, China; ^2^ Key Laboratory of Combined Multi-Organ Transplantation, Ministry of Public Health, China; ^3^ The Ophthalmology Center, The Second Affiliated Hospital, College of Medicine, Zhejiang University, Hangzhou, China; ^4^ Institute for Experimental Cancer Research, Comprehensive Cancer Center North, CAU, Kiel, Germany

**Keywords:** anatomy, microRNA, pancreatic cancer, recurrence

## Abstract

Different clinical presentations and prognoses have been implied between pancreatic head and body/tail cancers. We aimed to identify the prognostic relevance of primary tumor location in patients undergoing resection for pancreatic ductal adenocarcinoma (PDAC). Thirty-two pairs of patients with strictly matched early stage (II) pancreatic head and body/tail cancers were enrolled. The molecular feature of the two subtypes of PDAC was assessed on the level of miRNA expression. Out of the 64 patients, 34 (53.1%) had tumor recurrence after radical resection during the follow-up period (2.3 ± 0.8 years). Both overall and tumor-free survival were significantly higher in the patients with pancreatic body/tail cancer compared with those with pancreatic head cancer. Patient age and tumor location were the independent prognostic factors for tumor recurrence. A remarkably lower expression of miR-501-3p and higher expression of miR-375 were found and were further verified in pancreatic body/tail cancer tissues compared with pancreatic head cancer tissues. The low expression of miR-501-3p was significantly associated with a low risk of tumor recurrence. Both, subcutaneous and orthotopic PDAC mouse models presented highly invasive tumor phenotypes upon up-regulated miR-501-3p expression. An *in vitro* study showed that miR-501-3p promoted the invasiveness of PDAC cells possibly via suppressing E-cadherin. In summary, at resectable early stage, pancreatic body/tail cancer presents a less malignant phenotype associated with deregulation of miR-501-3p compared with pancreatic head cancer.

## INTRODUCTION

The tumor-node-metastasis (TNM) system has been widely used to stage tumors, predict prognosis and develop a therapeutic strategy. Recently, it has also been hypothesized that different tumor locations may be associated with varying clinical presentations and outcomes. Pioneering studies of colon cancer have demonstrated that right- and left-sided tumors exhibit different genetic, biological and demographic characteristics and risk factors [1].

With regard to the pancreas, tumor localization to the head has been found to be independently associated with local invasiveness and recurrence in pancreatic serous cystic neoplasms and intraductal papillary mucinous neoplasms [2, 3]. Branch duct intraductal papillary mucinous neoplasms typically arise in the head of the pancreas, while mucinous cystic neoplasms are common in the body or tail [4]. Furthermore, insulin-positive endocrine cells, which are present at a greater concentration in the tail of the pancreas compared with the head, could serve as a cell-of-origin of pancreatic ductal adenocarcinoma (PDAC) following oncogenic mutation inducement and pancreatic injury [5]. These findings indicate that the head, body, and tail of the pancreas may have different malignant potentials. In our recent review, we have concluded that pancreatic head cancer has a higher incidence, is easier to detect and has a better prognosis compared with pancreatic body/tail cancer [6]. However, early-stage tumors in the pancreas head may be associated with lower survival compared with those in the pancreas body/tail [6].

MicroRNAs (miRNAs) play powerful functional roles in controlling important cellular processes. Comparing with other potential biomarkers, miRNA can be found stabilized in body fluids and tissue samples, which makes it one of the most promising ways for earlier detection of cancer. Previous studies have identified the deregulation of a set of miRNAs in association with carcinogenesis in PDAC [7]. In the present study, we aimed to identify the prognostic relevance of primary tumor location in patients undergoing resection for PDAC, and to elucidate the epigenetic diversity between the two subtypes of PDAC by a comparison of miRNA expression profiles.

## RESULTS

### Tumor location is associated with tumor recurrence and patient survival

**Figure 1:**
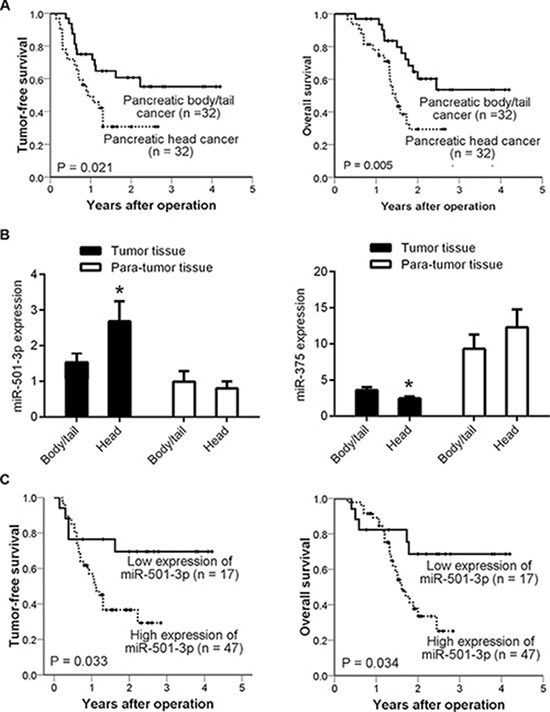
Low expression of miR-501-3p in pancreatic body/tail cancer, contributing to a low risk of tumor recurrence (**A**) Kaplan-Meier curves, showing both tumor-free survival and patient overall survival, which were higher in the patients with pancreatic body/tail cancer than in those with pancreatic head cancer; (**B**) Bars representing the significantly lower expression of miR-501-3p and higher expression of miR-375 in pancreatic body/tail cancer compared with pancreatic head cancer, as assessed by qRT-PCR; (**C**) Kaplan-Meier curves, presenting tumor-free survival and overall survival were significantly lower in the patients with high miR-501-3p expression than those with low expression. *P < 0.05 head vs. body/tail

Out of the 64 patients, 34 (53.1%) had tumor recurrence during the follow-up period (2.3 ± 0.8 years). The majority of cases with recurrence (24/34, 70.6%) occurred within 1 year after operation. The 1- and 2-year recurrence rates were significantly lower for the patients with pancreatic body/tail cancer compared with those with pancreatic head cancer (25.0% vs. 50.0%, *P* = 0.039; 37.5% vs. 65.6%, *P* = 0.024). Both overall survival and tumor-free survival were significantly higher in the patients with pancreatic body/tail cancer than in those with pancreatic head cancer (Figure 1A). In survival analysis, patient age > 60 years and tumor location at body/tail were found to be independent protective factors against tumor recurrence (Table 1).

**Table 1:** Cox regression analysis of influencing factors associated with tumor-free survival

	Univariate analysis		Multivariate analysis	
	*P*	Risk ratio (95% CI)	*P*	Risk ratio (95% CI)
Age (> 60 vs. ≤ 60 years)	0.036	0.452 (0.215–0.948)	0.033	0.443 (0.210–0.935)
Gender (male vs. female)	0.952	1.022 (0.505–2.068)		
CA 19-9 (positive vs. negative)	0.323	1.821 (0.555–5.977)		
Differentiation (moderate vs. moderate-poor/poor)	0.625	0.839 (0.415–1.697)		
Lymph node metastasis (yes vs. no)	0.712	0.881 (0.449–1.729)		
Location (body/tail vs. head)	0.025	0.449 (0.223–0.905)	0.023	0.441 (0.218–0.893)

CI, confidence interval; LT, liver transplantation; OR, odds ratio.

### Low recurrence risk of pancreatic body/tail cancer correlates with low expression of miR-501-3p

A miRNA microarray platform was used to compare miRNA profiles between randomly selected 4 pairs of strictly matched tumor tissues. The majority of miRNAs, including those associated with PDAC [7] (Supplementary Table 1), were not significantly differentially expressed between these two subtypes of PDAC. Of note, we found that 6 miRNAs were significantly differentially expressed (*P* < 0.05). Taking the fold-changes and rank sum differences into consideration, miR-501-3p was the only candidate for further study with a *P* < 0.05, a fold-change of > 2 and the largest rank sum difference. We also further assessed miR-375 because it has been reported to be a potent tumor suppressor in PDAC [8]. All 32 paired tumor tissues were used for subsequent validation. We verified the lower expression of miR-501-3p and higher expression of miR-375 in the pancreatic body/tail cancer tissues compared with the pancreatic head cancer tissues (Figure 1B).

Furthermore, the expression of miR-501-3p was significantly associated with tumor-free survival (*P* = 0.012, risk ratio [RR] = 1.164, 95% confidential interval [CI] = 1.034-1.311) and overall survival (*P* = 0.008, RR = 1.186, 95% CI = 1.046-1.346). In correlation analysis (Supplementary Table 2), higher miR-501-3p expression was significantly correlated with poorer differentiation (*r* = 0.272, *P* = 0.030). A cutoff value of miR-501-3p was then selected according to the receiver operating characteristic (ROC) curve and the low expression group showed significantly lower tumor recurrence rate (Supplementary Table 3) and higher tumor-free survival (Figure 1C) than the high expression group.

### MiR-501-3p promotes pancreatic cancer invasion *in vitro* and *in vivo*

**Figure 2:**
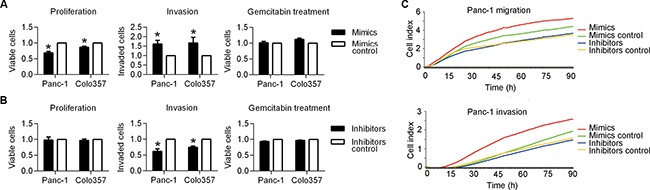
Biological effects of miR-501-3p on PDAC cells (**A**) The mimics group showed the significantly lower proliferation and higher invasiveness of both Panc-1 and colo357 cells compared with its negative control. (**B**) The inhibitors group presented significantly lower invasiveness of both Panc-1 and colo357 cells compared with its negative control. (**C**) A real-time invasion test using the xCelligence system, showing the much higher migration and invasion abilities of the mimics group compared with its negative control for Panc-1 cells. *P < 0.05.

An *in vitro* study was performed to evaluate the functional role of miR-501-3p in PDAC cells. A higher invasion rate in the mimics group (Panc1: *P* = 0.017, Colo357: *P* = 0.048) and a lower invasion rate in the inhibitors group (Panc1: *P* = 0.003, Colo357: *P* = 0.001), respectively, was found compared with their controls (Figure 2A and 2B). These results were further confirmed by a real-time migration and invasion test (Figure 2C).

**Figure 3:**
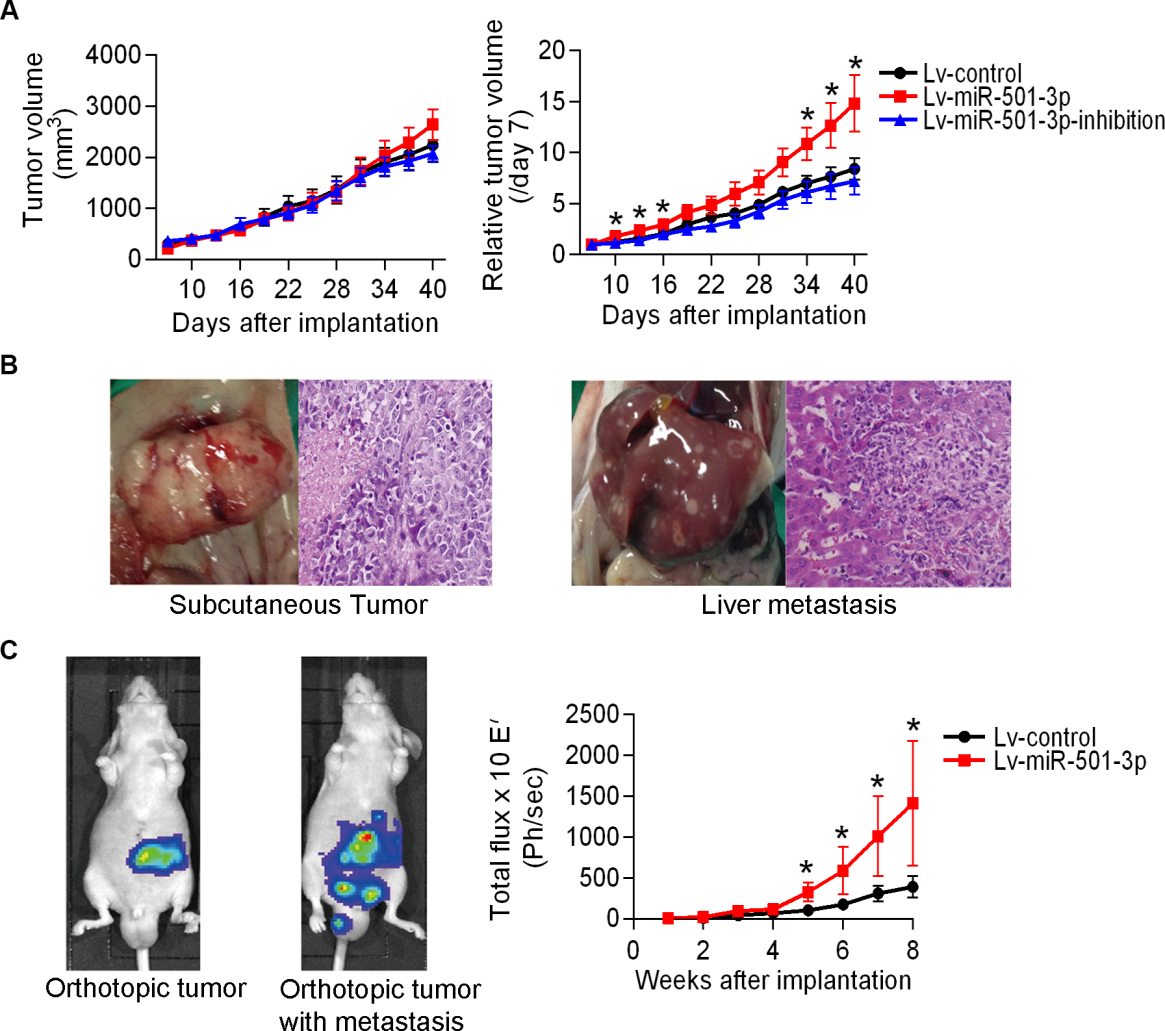
MiR-501-3p promotes tumor development in subcutaneous and orthotopic PDAC mouse models (**A**) A comparison of tumor size showed a significantly larger relative tumor volume for the Lv-miR-501-3p group compared with the Lv-control group. (**B**) Pathological examination confirmed liver metastases (right panel) of subcutaneous tumors (left panel) in the Lv-miR-501-3p group. (**C**) The primary tumor and abdominal metastatic burden in mice orthotopically implanted with tumor pieces. The Lv-miR-501-3p group showed a significantly higher tumor burden (reflected by total flux) than the Lv-control group. *P < 0.05.

A subcutaneous tumor mouse model was used to evaluate tumor growth by directly measuring tumor sizes. On the initial measurement day (7d), the mice in the Lv-miR-501-3p group (*n* = 7) had smaller tumor sizes compared with those in the Lv-control and Lv-miR-501-3p-inhibition groups (Figure 3A). After that, the mice in the Lv-miR-501-3p and Lv-miR-501-3p-inhibition groups showed increased and decreased tumor growth, respectively, compared with those in the Lv-control group (Figure 3A). However, there were no significant differences in tumor volume (TV) between the experimental and control groups. In contrast, the Lv-miR-501-3p group presented a significantly elevated relative tumor volume (RTV) curve compared with that of the Lv-control group (Figure 3B). At the end of the experiment, the mice were sacrificed. Intact capsules and central necrosis were found in all tumors. Two out of 6 mice had liver metastasis in the Lv-miR-501-3p group (Figure 3B), while no metastasis was found in the other two groups.

Furthermore, using an *in vivo* imaging system, tumor burden was recorded in an orthotopic PDAC model (Figure 3C). Compared with the Lv-control group (*n* = 8), the Lv-miR-501-3p group demonstrated a higher tumor burden and faster tumor development (Figure 3C). The first tumor metastasis event was observed in the Lv-miR-501-3p group at 5 weeks after implantation (Figure 3C). At 6 weeks, a mouse in the Lv-miR-501-3p group died, possibly due to a high tumor burden. At the end of the experiment, a total of 3 mice had tumor metastasis, and 1 mouse had died in the Lv-miR-501-3p group. In contrast, no mouse experienced tumor metastasis or died in the Lv-control group.

In addition, the expression of miR-501-3p in tumor tissues was evaluated to confirm the efficiency of lentivirus infection (Supplementary Figure 1). The proliferation and invasion capabilities of Panc-1 cells infected with lentivirus were also assessed and were shown to be consistent with those of Panc-1 cells transfected with miRNA mimics and inhibitors (Supplementary Figure 2).

### MiR-501-3p down-regulates E-cadherin in pancreatic cancer

**Figure 4:**
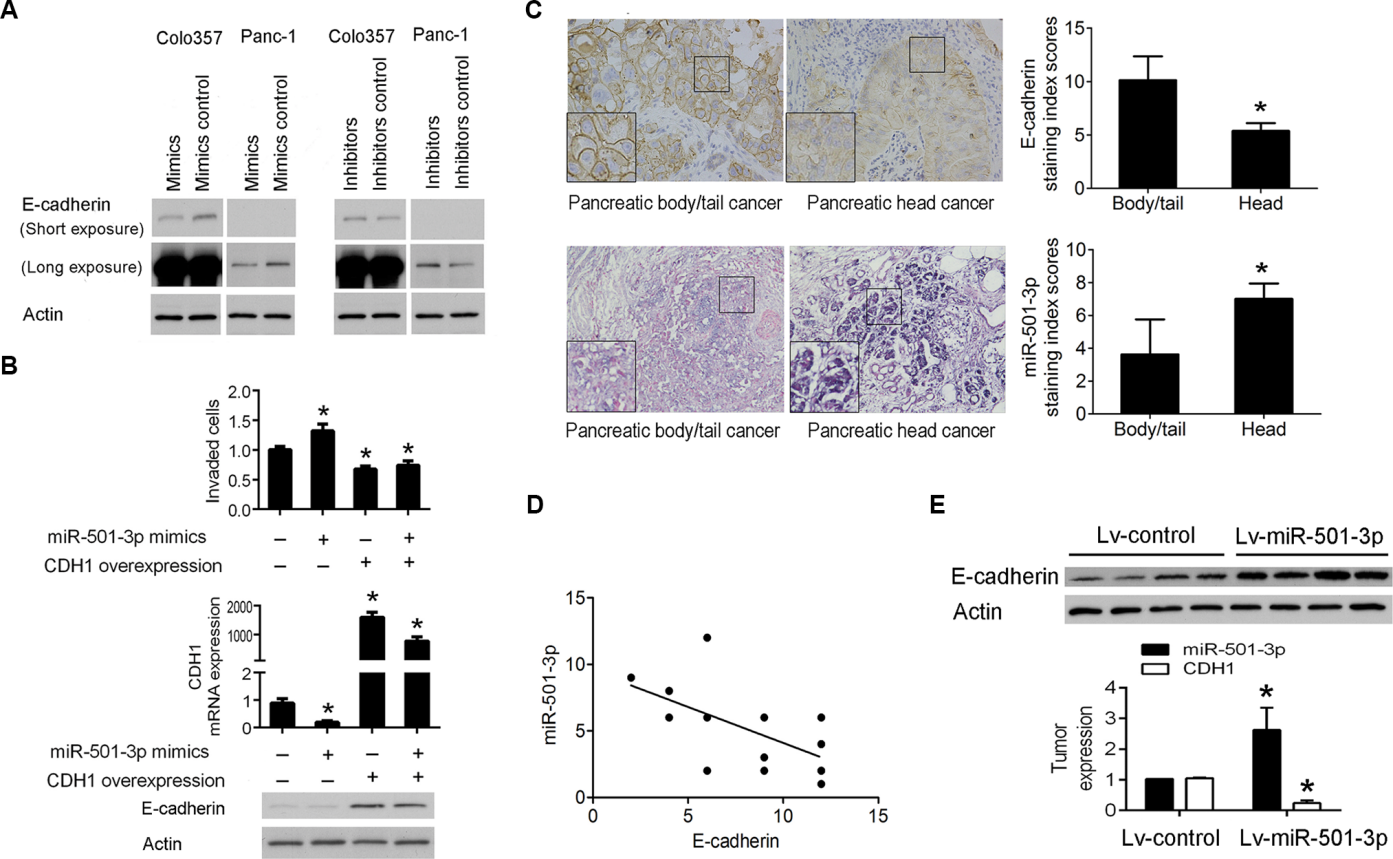
MiR-501-3p down-regulates the expression of E-cadherin (**A**) The expression of E-cadherin was lower in the mimics group and higher in the inhibitors group, respectively, compared with their negative controls in both Panc-1 and Colo357 cells. (**B**) The enforced expression of E-cadherin reduces the miR-501-3p-enhanced invasiveness in Panc-1 cells. (**C**) The higher and lower expression of E-cadherin and miR-501-3p, respectively, in human pancreatic body/tail cancer tissues compared with pancreatic head cancer tissues (400×). (**D**) Linear correlation analysis showed a negative correlation between the expression of E-cadherin and miR-501-3p. (**E**) In the orthotopic PDAC model, Lv-miR-501-3p group showed higher expression of miR-501-3p and lower expression of E-cadherin mRNA and protein in the tumor tissue than the Lv-control group.*P< 0.05.

To reveal the underlying mechanism of miR-501-3p-induced invasion, an antibody microarray was used to analyze differential protein expression between Panc-1 cells transfected with either miR-501-3p mimics or its control (Supplementary Table 4). But the significantly different expressed proteins were not predicted to be direct potential targets of miR-501-3p using open-source software. Yet, E-cadherin as a well-studied one involved in cancer invasion has an interaction network with miR-501-3p (Supplementary Figure 3), and thus was chosen for further experiments. The high and low E-cadherin-expressing cell lines (Colo357 and Panc-1, respectively) were used (Supplementary Figure 4). We found reduced E-cadherin protein expression in the mimics group and its increased expression in the inhibitors group compared with their respective controls in both cell lines (Figure 4A). Furthermore, we manipulated the E-cadherin expression and found the enforced expression of E-cadherin could rescue the miR-501-3p effects in Panc-1 cells (Figure 4B).

Using immunohistochemistry analysis, we found significantly high and low expressed E-cadherin and miR-501-3p, respectively, in pancreatic body/tail cancers than pancreatic head cancers (Figure 4C). There was a significant negative correlation between the expression of E-cadherin and miR-501-3p (*r* = –0.596, *P* = 0.015, Figure 4D). In the orthotopic PDAC model, Lv-miR-501-3p group presented higher expression of E-cadherin in the tumor tissue than the Lv-control group (Figure 4E).

## DISCUSSION

To the best of our knowledge, the present study is the first comparison of miRNA expression patterns between strictly matched early stage pancreatic body/tail and head cancers. The patients with early stage pancreatic body/tail cancer showed increased survival compared with the paired patients with pancreatic head cancer, which is consistent with a previous report [9]. In addition, we found that the patients with early stage pancreatic body/tail cancer had a lower tumor recurrence rate after curative resection. Although the detailed molecular mechanism of tumor recurrence is still unclear, a high tumor recurrence rate could signify more malignant potential, especially if the same surgeon has performed all operations and the tumors are of the same stage and histological differentiation. These clinical results indicate that pancreatic body/tail cancer might be less malignant than pancreatic head cancer.

Because both pancreatic head and body/tail cancers are essentially PDAC, it is not surprising that the expression levels of the majority of miRNAs were similar between these two subtypes. However, two miRNAs (miR-501-3p and miR-375) were significantly differentially expressed between the two subtypes. Studies have demonstrated a down-regulation of miR-375 in PDAC [7]. It can act as a tumor suppressor and targets several oncogenes, including *PDK1* and *JAK2*, resulting in decreases in cancer cell growth, viability and invasiveness [10, 11]. Unlike miR-375, miR-501-3p has not yet been well studied in human cancers. Previous study showed an overexpression of miR-501-3p in non-functioning pituitary adenomas [12], malignant melanoma [13], compared with normal tissues. A clinically relevant study found that serum miR-501-3p was elevated in patients with early stage PDAC compared with those with chronic pancreatitis or normal controls [14]. Our results demonstrated that miR-501-3p promoted PDAC recurrence after surgery. It indicates that miR-501-3p might be a potential biomarker for PDAC carcinogenesis or tumor development [14]. In addition, from the clinical characteristics analysis, we found that high miR-501-3p expression maybe linked with poor tumor differentiation but not lymphatic metastasis. We suggest that pancreatic body/tail cancer maybe characterized by the lower expression of miR-501-3p, which contributes to a lower recurrence risk, compared with pancreatic head cancer.

Both the *in vitro* and *in vivo* studies confirmed this hypothesis. Furthermore, miR-501-3p possibly promoted cancer invasion via down-regulating E-cadherin, which is well known as a transmembrane protein localized to the adherence junctions of the epithelial cell basolateral surface that plays a key role in epithelial morphology maintenance. The loss of E-cadherin expression is a well-recognized marker of EMT, and it promotes PDAC progression, invasion and metastasis [15]. Taken together, our results indicate that the lower expression of miR-501-3p in pancreatic body/tail cancer might be associated with the higher expression of E-cadherin, and subsequently, a less invasive/metastasis phenotype compared with pancreatic head cancer.

A limitation of this clinical study is the relatively small cohort assessed. Nevertheless, considering the relatively low incidence of pancreatic body/tail cancer and more importantly, the difficulty in strictly matching between the two groups, this study should have value. On the other hand, since stringent selection criteria were applied in the initial screening step, a selection bias cannot be ruled out. Another possible limitation is ethnic in nature. Our results should be verified in Western cohorts.

In summary, this study is the first to compare pancreatic head and body/tail cancers using strictly matched early stage PDAC tissue samples, and show the prognostic significance of tumor location in PDAC. We also demonstrated that miR-501-3p promotes the invasiveness of PDAC cells, possibly via regulating the expression of E-cadherin. These findings confirm the importance of subsite division and support the development of precision medicine.

## MATERIALS AND METHODS

### Tissue samples

Patients with newly diagnosed early stage (I–II) PDAC were considered eligible for this study. The research protocol was approved by the ethical committee of the First Affiliated Hospital, Zhejiang University and strictly followed the Declaration of Helsinki. Tumor samples were obtained directly during surgery. Thirty-two matched fresh frozen tissue samples from patients with pancreatic head cancer and body/tail cancer were selected. These 64 patients were strictly matched by gender, age (± 10 years), CA 19-9 (–/+), TNM stage, and histological differentiation (Supplementary Table 5).

### MicroRNA microarray

Microarray analysis was performed using an Agilent Human miRNA Microarray Kit (8 × 15 k), version 14.0 (design ID: 31945) according to the manufacturer's protocols. Total RNA was extracted and purified using a mirVana™ miRNA Isolation Kit (Ambion, Austin, TX, USA), and the RIN number was determined to assess RNA integrity with an Agilent Bioanalyzer 2100 (Agilent Technologies, Santa Clara, CA, USA). The data were analyzed with online SBC analysis system (http://www.shbio.com/sas.html, ShanghaiBio Corp., Shanghai, China) and uploaded to Gene Expression Omnibus (GSE83496). The *t-test*, fold change and Benjamini-Hochberg's false discovery rate methods were used.

### Real-time PCR

Real-time PCR was performed using a Real-time PCR System 9700 (Applied Biosystems, Carlsbad, CA, USA) and SDS 2.1 software (Applied Biosystems) as we described previously [16].

### Cell culture and transfection

Human PDAC cell lines (Capan-1, Capan-2, Panc-1, PancTu-1, Panc89, BxPc-3 and Colo357) and culture condition had been described previously [17]. MiR-501-3p mimics, inhibitors or their negative controls were purchased from Life Technologies, Darmstadt, Germany in order to positively or negatively influence the respective miRNA level. Plasmids were synthesized by GenePharma, Shanghai, China. Transfection was performed according to the manufacturer's protocol [16].

### Cell viability and invasion assay

Cell viability was examined by crystal violet staining (Sigma-Aldrich Chemie GmbH, München, Germany). Cell invasion was assessed using collagen I-coated ThinCerts™ Cell Culture Inserts (8 μm, Greiner Bio-One, Frickenhausen, Germany) and xCelligence DP system. The procedure has been described previously [16, 18].

### Antibody array

A Signaling Explorer Antibody Array (SET100) was purchased from Full Moon Biosystems, Inc. (Sunnyvale, CA, USA) and processed according to the suggested protocol. Briefly, proteins were labeled with Biotin/DMF and placed on preblocked microarray slides. After washing, proteins were detected using Cy3-conjugated streptavidin. Data were captured using a ScanArray Gx scanner (GenePix 4000B, Axon Instruments, Foster city, Calif., USA) and GenePix Pro 6.0 software (Axon Instruments). The expression of each protein contained in the array was normalized to that of β-actin and GAPDH, respectively. Different expressed proteins were selected by > 2.0-fold variations between two samples.

### Western blot analysis

Western Blot analysis was performed as described previously [16]. The primary antibodies used were: anti-human E-cadherin antibody (1:2500, R&D systems, Wiesbaden, Germany), and anti-β actin antibody (1:50000, Sigma-Aldrich Chemie GmbH, München, Germany).

### Dual luciferase activity assay

Double-stranded oligonucleotides corresponding to the wild-type (WT-3′-UTR) or mutant (MUT-3′-UTR) miR-501-3p binding sites in the 3′-UTRs of the potential target genes were synthesized (GenePharma, Shanghai, China) and ligated into apsiCHECK™-2 Vector (Promega, Madison, WI, USA). The detailed procedure has been described previously [19].

### Immunohistochemistry and miRNA *in situ* hybridization

Immunohistochemistry was performed and scored as we described previously [20]. Tissue sections were deparaffinized, rehydrated, and then treated with 40 μg/ml proteinase K for 20 min at 37°C, fixed with 4% formaldehyde in PBS, rinsed twice in 0.13 M 1-methylimidazole and refixed with 1-ethyl-3-(3-dimethylaminopropyl) carbodiimide. Slides were prehybridized in a hybridization oven for 30 min in hybridization buffer with 500 μg/ml yeast tRNA, 50% formamide, 2 × SSC, 50 μg/ml heparin, and 0.1% Tween 20, with the pH adjusted to 6. The slides were then hybridized for 1 h with 200 nM double-digoxigenin LNA-modified probes (Exiqon, Vedbaek, Denmark) for miR-501-3p. Next, the slides were stringently washed in 2 × SSC, blocked with 2% BSA for 30 min and incubated with anti-DIG-AP at 4°C overnight. After two washes with 0.1% TBST, miRNA signals were detected with a BCIP/NBT system (Merck, Whitehouse Station, NJ, USA).

### Pancreatic cancer xenotransplant models

The animal use protocol was reviewed and approved by Institutional Animal Care and Use Committee of the First Affiliated Hospital, Zhejiang University and was in accordance with the Declaration of Helsinki. Four- to six-week-old male BALB/c-nu/nu mice were obtained from Charles River (Kanagawa, Japan). Hsa-miR-501-3p-recombinant lentivirus and control vectors were constructed by GeneChem Corp. (Shanghai, China). Panc-1/Luc cells were infected with lentivirus (Lv-miR-501-3p, Lv-miR-501-3p-inhibition, and Lv-control) at a multiplicity of infection of 2. Detailed information on the lentiviruses is provided in Supplemental Table 6.

A subcutaneous tumor mouse model was established by subcutaneous implantation of 1 × 10^7^ cells into the backs of mice. TV was measured at different time points (initially at day 7) and was calculated as TV (mm^3^) = (length × width^2^)/2. RTV was calculated as TV (day n)/TV (day 7) and was used as a parameter for tumor progression. Subcutaneous tumors were also resected under aseptic conditions after growing to a certain volume. Viable tumor tissues were cut into small pieces of 1 mm^3^. The tumor pieces were then implanted into the mouse pancreases. Tumor growth and metastasis were monitored every week by bioluminescence imaging using an *in vivo* imaging system (AMI-1000; Spectral Instrument Imaging, Tucson, AZ, USA).

### Statistical analysis

Statistical analyses were performed using SPSS 13.0 for Windows and SBC analysis system. A *P* < 0.05 was considered statistically significant. The Kolmogorov-Smirnov test was used to check for normal distribution of measurements. Quantitative variables were expressed as the mean ± SD or the median. Categorical variables were presented as values and percentages. The paired-sample *t* test, Wilcoxon-Mann-Whitney test, one-way ANOVA and chi-square test were used to compare quantitative and categorical variables, respectively. The Kaplan-Meier method and log-rank test were used for survival comparisons. Cox regression analysis was used to determine the risk factors for tumor recurrence after curative surgical resection. The cutoff value was determined using a ROC curve.

## SUPPLEMENTARY MATERIALS FIGURES AND TABLES




