# Supplementary material for: The prognostic relevance of primary tumor location in patients undergoing resection for pancreatic ductal adenocarcinoma
Source: Oncotarget. 2017 Jan 20;8(9):15159–67. doi: 10.18632/oncotarget.14768 (PMC5362475; doi:10.18632/oncotarget.14768)
Supplement: Supplementary file 1 [file oncotarget-08-15159-s001.pdf]

The prognostic relevance of primary tumor location in patients undergoing resection for pancreatic ductal adenocarcinoma

Supplementary Materials

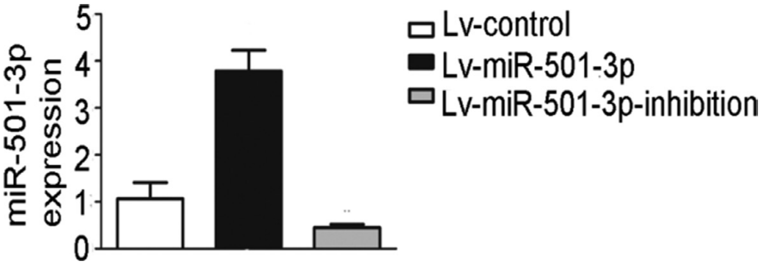

Supplementary Figure 1: The expression of miR-501-3p in different mice tumors. **P* < 0.05 compared with Lv-control.

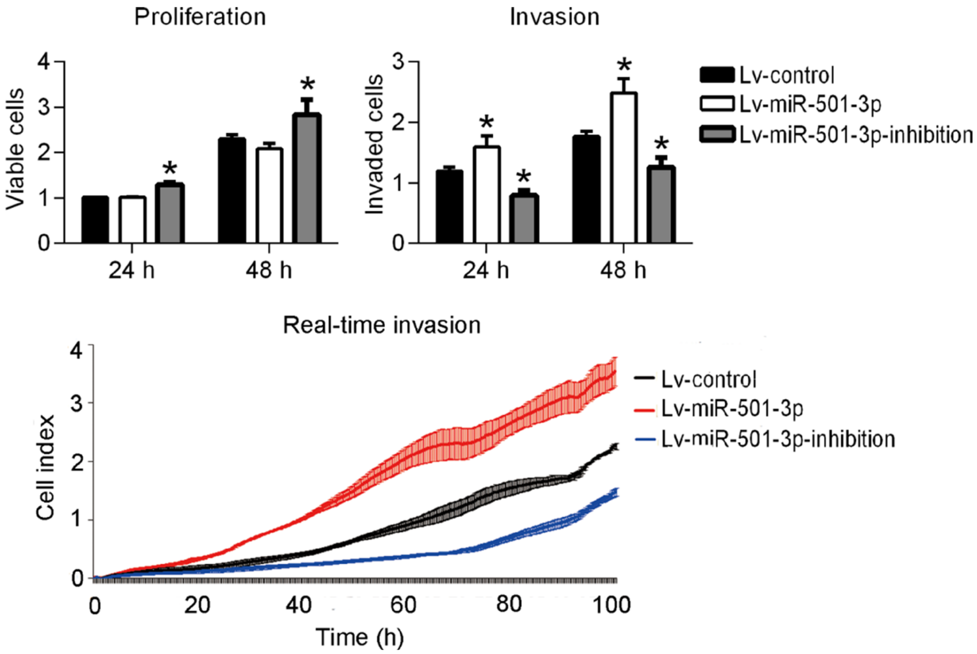

Supplementary Figure 2: The effect of miR-501-3p on the proliferation and invasion capability changes of Panc-1 cells infected with different lentivirus. **P* < 0.05 compared with Lv-control.

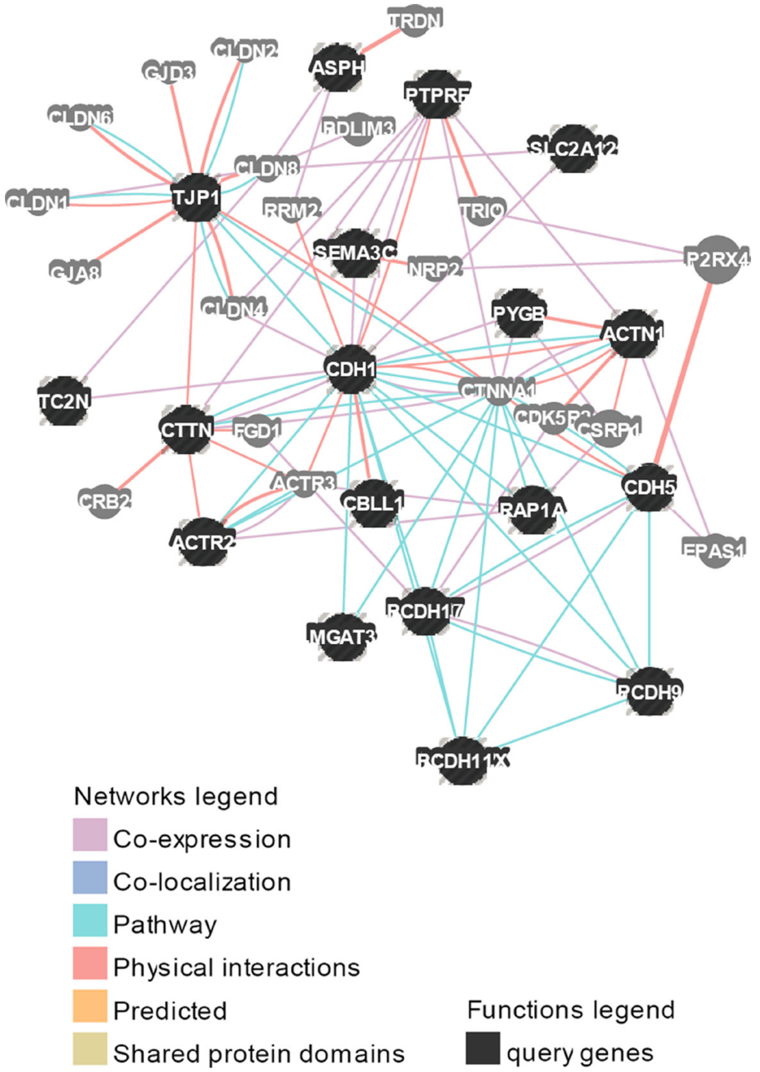

Supplementary Figure 3: MiR-501-3p potential targets interact with *CDH1* (encoding E-cadherin). Network image was constructed using GeneMANIA database (application version 3.1.1).

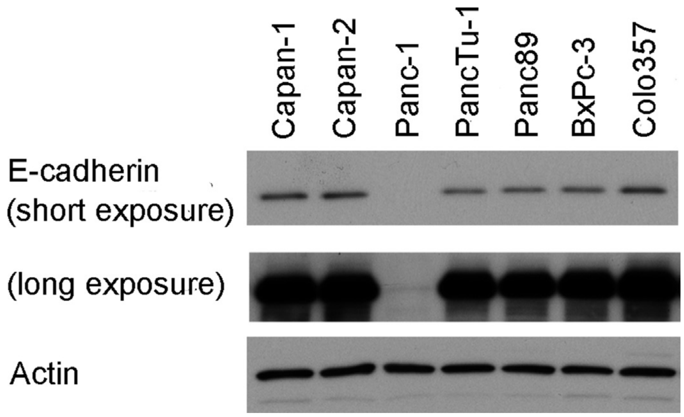

Supplementary Figure 4: The expression of E-Cadherin in different PDAC cell lines.

Supplementary Table 1: The regulation of pancreatic cancer associated miRNAs in pancreatic body/tail cancer compared with pancreatic head cancer. See Supplementary_Table_1

Supplementary Table 2: Correlation between miR-501-3p expression and tumor characteristics

	Correlation analysis	
	**r**	***P* value**
T stage	0.039	0.762
Lymph node metastasis	0.046	0.718
Differentiation	0.272	0.030
CA 19-9	–0.044	0.732

Supplementary Table 3: The comparison between patients with low and high expression of tumor miR-501-3p

	**Low expression of miR-501-3p (*n* = 17)**	**High expression of miR-501-3p (*n* = 47)**	***P***
Gender			0.272
Male (*n* = 42)	13 (76.5%)	29 (61.7%)	
Female (*n* = 22)	4 (13.5%)	18 (38.3%)	
Age			0.529
> 60 years (*n* = 26)	8 (47.1%)	18 (38.3%)	
≤ 60 years (*n* = 38)	9 (52.9%)	29 (61.7%)	
CA 19-9			0.693
Positive (*n* = 58)	15 (88.2%)	43 (91.5%)	
Negative (*n* = 6)	2 (11.8%)	4 (8.5%)	
T stage			0.693
2 (*n* = 6)	2 (11.8%)	4 (8.5%)	
3 (*n* = 58)	15 (88.2%)	43 (91.5%)	
Lymph node metastasis			0.396
Yes (*n* = 32)	10 (58.5%)	22 (46.8%)	
No (*n* = 32)	7 (41.2%)	25 (53.2%)	
Differentiation			0.101
Moderate-poor/poor (*n* = 44)	9 (52.9%)	35 (74.5%)	
Moderate (*n* = 20)	8 (47.1%)	12 (25.5%)	
Tumor recurrence			0.022
Yes (*n* = 34)	5 (29.4%)	29 (61.7%)	
No (*n* = 30)	12 (70.6%)	18 (38.3%)	
Death			0.067
Yes (*n* = 31)	5 (29.4%)	26 (55.3%)	
No (*n* = 33)	12 (70.6%)	21 (44.7%)	

Supplementary Table 4: Regulation of proteins in Panc-1 cells transfected with miR-501-3p Mimics compared with its negative control

	β-actin (internal control)		GAPDH (internal control)	
	Regulation	Mimics/control	Regulation	Mimics/control
DP-1	Up	5.89	Up	7.60
Desmin	Up	3.57	Up	4.60
RCBTB1	Up	2.46	Up	3.17
COX1	Up	2.40	Up	3.09
RIT1	Up	2.11	Up	2.72
GAD1/2	Up	2.06	Up	2.65
Pax-5	Up	2.03	Up	2.61
ARSI	/	/	Up	2.01
mGluR2/3	/	/	Up	2.11
Keratin 15	/	/	Up	2.22
Retinoic Acid Receptor β	/	/	Up	2.27
MMP-10	/	/	Up	2.38
Catenin-γ	/	/	Up	2.48
CA125	Down	0.13	Down	0.16
eNOS	Down	0.28	Down	0.36
MAPK 11	Down	0.28	Down	0.36
NKX2.5	Down	0.30	Down	0.39
SND1/P100	Down	0.31	Down	0.40
CD37	Down	0.32	Down	0.41
Nucleophosmin (NPM)	Down	0.33	Down	0.42
GPR174	Down	0.33	Down	0.43
MAP4K4	Down	0.33	Down	0.43
Cytochrome b561 D1*	Down	0.34	Down	0.43
Tyk2	Down	0.35	Down	0.46
cAMP	Down	0.35	Down	0.46
GPR175	Down	0.37	Down	0.48
Parkin	Down	0.37	Down	0.48
INHA (Inhibin α)*	Down	0.37	Down	0.48
CD44	Down	0.39	Down	0.50
E-cadherin	Down	0.39	Down	0.50
Claudin 5	Down	0.39	/	/
Calreticulin	Down	0.39	/	/
SOD1	Down	0.41	/	/
CSF2 (GM-CSF)	Down	0.42	/	/
SUMO2/3 (Cleaved-Gly93)	Down	0.42	/	/
NCOA3	Down	0.42	/	/
Actin-alpha-1	Down	0.43	/	/
Myostatin*	Down	0.44	/	/
Histone H3 (Acetyl-Lys23)	Down	0.45	/	/
MUM1	Down	0.45	/	/
IL-1 beta	Down	0.45	/	/
CD14	Down	0.46	/	/
CASP3 (p17,Cleaved-Asp175)	Down	0.48	/	/
AGR3	Down	0.48	/	/
MMP23 (Cleaved-Tyr79)	Down	0.48	/	/
MPS1	Down	0.48	/	/
C-Kit	Down	0.48	/	/
Angiopoietin-2	Down	0.48	/	/
AKT2*	Down	0.49	/	/
TSH	Down	0.49	/	/
FGF-1	Down	0.49	/	/
Cullin 1	Down	0.49	/	/
TAF5L*	Down	0.50	/	/

*potential target genes predicted by TargetScanHuman (http://www.targetscan.org).

Supplementary Table 5: Tumor samples used for analysis

	Gender	Age (year)	CA 19-9 (ng/ml)	Histological differentiation	TNM staging
Paired 1	male	73	49.1	Moderate-poor	T3N0M0 (II A)
	male	81	240.3	Moderate-poor	T3N0M0 (II A)
Paired 2	female	50	4481.2	Moderate-poor	T2N1M0 (II B)
	female	42	173	Moderate-poor	T2N1M0 (II B)
Paired 3	female	61	995	Moderate	T3N0M0 (II A)
	female	53	53.1	Moderate	T3N0M0 (II A)
Paired 4	male	63	893.7	poor	T3N1M0 (II B)
	male	70	961	poor	T3N1M0 (II B)
Paired 5	female	55	572	Moderate	T3N1M0 (II B)
	female	50	951	Moderate	T3N1M0 (II B)
Paired 6	male	52	471	Moderate-poor	T3N0M0 (II A)
	male	61	11246.5	Moderate-poor	T3N0M0 (II A)
Paired 7	male	55	1598	Moderate-poor	T3N1M0 (II B)
	male	56	71.3	Moderate-poor	T3N1M0 (II B)
Paired 8	female	59	3	Moderate-poor	T3N0M0 (II A)
	female	55	2	Moderate-poor	T3N0M0 (II A)
Paired 9	female	41	54.4	Moderate-poor	T3N1M0 (II B)
	female	39	104.3	Moderate-poor	T3N1M0 (II B)
Paired 10	female	50	296.2	Moderate-poor	T3N0M0 (II A)
	female	43	1196.9	Moderate-poor	T3N0M0 (II A)
Paired 11	female	63	1259.6	Moderate-poor	T3N0M0 (II A)
	female	58	3557	Moderate-poor	T3N0M0 (II A)
Paired 12	female	61	3298.1	Moderate	T3N0M0 (II A)
	female	64	200.4	Moderate	T3N0M0 (II A)
Paired 13	female	54	75.1	poor	T3N0M0 (II A)
	female	59	748.5	poor	T3N0M0 (II A)
Paired 14	male	57	647.1	Moderate-poor	T3N0M0 (II A)
	male	56	232	Moderate-poor	T3N0M0 (II A)
Paired 15	male	67	3	Moderate-poor	T3N0M0 (II A)
	male	60	3	Moderate-poor	T3N0M0 (II A)
Paired 16	male	69	12000	Moderate	T3N0M0 (II A)
	male	75	7753.6	Moderate	T3N0M0 (II A)
Paired 17	male	62	22.7	Moderate-poor	T3N0M0 (II A)
	male	57	10	Moderate-poor	T3N0M0 (II A)
Paired 18	male	75	1549.5	Moderate-poor	T3N0M0 (II A)
	male	79	12000	Moderate-poor	T3N0M0 (II A)
Paired 19	male	62	1973.7	Moderate	T3N0M0 (II A)
	male	52	724.7	Moderate	T3N0M0 (II A)
Paired 20	male	60	500.6	Moderate	T3N0M0 (II A)
	male	56	180.6	Moderate	T3N0M0 (II A)
Paired 21	male	70	583.7	poor	T3N0M0 (II A)
	male	78	1090.7	poor	T3N0M0 (II A)
Paired 22	female	58	221.3	Moderate-poor	T3N1M0 (II B)
	female	46	132.1	Moderate-poor	T3N1M0 (II B)
Paired 23	female	51	160.1	Moderate	T2N1M0 (II B)
	female	53	74	Moderate	T2N1M0 (II B)
Paired 24	male	45	335.9	Moderate-poor	T3N1M0 (II B)
	male	47	605.1	Moderate-poor	T3N1M0 (II B)
Paired 25	male	51	1086.5	Moderate-poor	T3N1M0 (II B)
	male	60	245.2	Moderate-poor	T3N1M0 (II B)
Paired 26	male	58	341.9	Moderate-poor	T3N1M0 (II B)
	male	58	718.6	Moderate-poor	T3N1M0 (II B)
Paired 27	male	59	760	Moderate-poor	T3N1M0 (II B)
	male	69	1528.3	Moderate-poor	T3N1M0 (II B)
Paired 28	male	64	3432.7	Moderate-poor	T3N1M0 (II B)
	male	64	1518.8	Moderate-poor	T3N1M0 (II B)
Paired 29	male	67	358.9	Moderate-poor	T2N1M0 (II B)
	male	68	136.9	Moderate-poor	T2N1M0 (II B)
Paired 30	male	53	300	Moderate	T3N1M0 (II B)
	male	48	602.9	Moderate	T3N1M0 (II B)
Paired 31	male	64	309.7	Moderate	T3N1M0 (II B)
	male	59	700	Moderate	T3N1M0 (II B)
Paired 32	male	80	1142.1	Moderate	T3N1M0 (II B)
	male	75	271.3	Moderate	T3N1M0 (II B)

Supplementary Table 6: The detailed information about control, over-expression and inhibition lentivirus

Virus	Target gene	Target sequence	Vector	Component sequence	Titer (TU/ml)
Lv-mir-501-3p	hsa-mir-501	5′-GCUCUUCCUCUCUAA UCCUUUGUCCCUGGGUG AGAGUGCUUUCUGAAUGC AAUGCACCCGGGCAAGG AUUCUGAGAGGGUGAGC-3′	GV254 http://www.genechem.com.cn/Zaiti.aspx?zt=GV254	Ubi-EGFP-MCS-IRES-Puromycin	5E+8
Lv-mir-501-3p-inhibition	hsa-miR-501-3p	5′-AGAATCCTTGCCCGGGT GCATT-3′ (miRNA mature reverse complement sequence)	GV280 http://www.genechem.com.cn/Zaiti.aspx?zt=GV280	hU6-MCS-Ubiquitin-EGFP-IRES-puromycin	8E+8
Lv-control (CON137)	NC	5′- TTCTCCGAACGTGTCACGT-3′	GV280 http://www.genechem.com.cn/Zaiti.aspx?zt=GV280	H1-NC-CMV-EGFP	1E+9
