# Supplementary material for: The prognostic relevance of primary tumor location in patients undergoing resection for pancreatic ductal adenocarcinoma
Source: Oncotarget. 2017 Jan 20;8(9):15159–67. doi: 10.18632/oncotarget.14768 (PMC5362475; doi:10.18632/oncotarget.14768)
Supplement: Supplementary file 2 [file oncotarget-08-15159-s002.doc]

Supplementary Table 1 The regulation of pancreatic cancer associated miRNAs in pancreatic body/tail cancer compared with pancreatic head cancer

| miRNA | *P* value | Fold change | Regulation | | Expression | |
| --- | --- | --- | --- | --- | --- | --- |
|  |  |  | Body/tail | In PDAC | Head | Body/tail |
| hsa-miR-126* | 0.010409 | 1.497677 | up |  | 5.083503 | 5.666229 |
| hsa-miR-455-3p | 0.015982 | 2.201055 | up |  | 5.655185 | 6.79338 |
| hsa-miR-501-3p | 0.019877 | 14.17093 | down |  | 2.385106 | -1.43976 |
| hsa-miR-320d | 0.020129 | 1.211291 | down |  | 8.939015 | 8.66247 |
| hsa-miR-320b | 0.043105 | 1.154092 | down | Up | 8.651562 | 8.444804 |
| hsa-miR-375 | 0.045640 | 5.612237 | up | down | 5.595712 | 8.084288 |
| hsa-miR-146b-5p | 0.074065 | 1.681926 | up | up | 8.300115 | 9.050231 |
| hsa-miR-148a | 0.076712 | 1.66357 | up | down | 8.791904 | 9.526186 |
| hsa-miR-10b | 0.114725 | 1.345653 | up | up | 7.734763 | 8.16307 |
| hsa-miR-146b-3p | 0.132901 | 1.114185 | up | up | -3.17074 | -3.01476 |
| hsa-miR-190 | 0.132901 | 1.114185 | up | up | -3.17074 | -3.01476 |
| hsa-miR-220a | 0.132901 | 1.114185 | up | up | -3.17074 | -3.01476 |
| hsa-miR-220b | 0.132901 | 1.114185 | up | up | -3.17074 | -3.01476 |
| hsa-miR-301b | 0.132901 | 1.114185 | up | up | -3.17074 | -3.01476 |
| hsa-miR-424 | 0.148204 | 1.727851 | up | up | 7.863314 | 8.652293 |
| hsa-miR-21 | 0.203332 | 1.126374 | up | up | 15.59998 | 15.77166 |
| hsa-miR-142-5p | 0.226626 | 1.823401 | up | down | 6.074466 | 6.941097 |
| hsa-miR-155 | 0.231053 | 1.420632 | up | up | 7.461748 | 7.968281 |
| hsa-miR-139-3p | 0.238155 | 1.480683 | up | down | 3.542734 | 4.108997 |
| hsa-miR-148b | 0.240651 | 1.357972 | up | down | 6.37236 | 6.813814 |
| hsa-let-7d | 0.265628 | 1.124965 | up | up | 9.977742 | 10.14762 |
| hsa-miR-142-3p | 0.300062 | 1.459817 | up | down | 9.966391 | 10.51218 |
| hsa-miR-186 | 0.315269 | 1.293223 | up | up | 6.044224 | 6.415195 |
| hsa-miR-125a-5p | 0.336444 | 1.539021 | up | up | 7.296905 | 7.918917 |
| hsa-miR-345 | 0.336664 | 6.250811 | down | down | -0.37071 | -3.01476 |
| hsa-miR-100 | 0.339273 | 1.266009 | down | up | 9.68679 | 9.346502 |
| hsa-miR-212 | 0.345962 | 1.213576 | up | up | 3.896251 | 4.175515 |
| hsa-miR-99a | 0.346617 | 1.321411 | down | up | 9.234546 | 8.832466 |
| hsa-miR-217 | 0.353879 | 4.632326 | up | down | -3.17074 | -0.95901 |
| hsa-miR-139-5p | 0.366812 | 5.930201 | up | down | -1.13349 | 1.434589 |
| hsa-miR-99b | 0.377206 | 1.456922 | up | up | 6.921114 | 7.464037 |
| hsa-miR-95 | 0.381705 | 4.517441 | up | up | 3.342103 | 5.517609 |
| hsa-miR-92b | 0.435211 | 3.030231 | up | up | 1.836418 | 3.435846 |
| hsa-miR-301a | 0.436055 | 1.886369 | up | up | 3.992251 | 4.907863 |
| hsa-miR-199a-3p | 0.457781 | 1.272802 | up | up | 11.92264 | 12.27065 |
| hsa-miR-181a | 0.463916 | 1.28108 | up | up | 9.070984 | 9.428344 |
| hsa-miR-145 | 0.476132 | 1.298614 | down | up | 10.74572 | 10.36875 |
| hsa-let-7i | 0.485352 | 1.118322 | up | up | 11.6396 | 11.80094 |
| hsa-miR-223 | 0.514289 | 1.628822 | up | up | 10.28607 | 10.9899 |
| hsa-miR-15b | 0.517299 | 1.1142 | up | up | 9.94014 | 10.09615 |
| hsa-miR-205 | 0.529800 | 1.14686 | down | up | -2.81707 | -3.01476 |
| hsa-miR-376a | 0.531236 | 1.189124 | up | up | 6.985436 | 7.235335 |
| hsa-miR-96 | 0.551678 | 1.743802 | down | down | 6.748685 | 5.946449 |
| hsa-miR-125b | 0.552814 | 1.101894 | down | up | 12.1143 | 11.97432 |
| hsa-miR-10a | 0.558727 | 1.316333 | up | up | 9.298406 | 9.69493 |
| hsa-miR-181b | 0.561575 | 1.305739 | up | up | 6.701516 | 7.086381 |
| hsa-miR-196a | 0.574712 | 3.273563 | up | up | 0.89446 | 2.605321 |
| hsa-miR-92a | 0.593379 | 1.139964 | down | up | 8.240348 | 8.051362 |
| hsa-miR-194 | 0.595127 | 2.105559 | down | up | 8.641068 | 7.566865 |
| hsa-miR-130b | 0.642252 | 1.211653 | up | down | 6.15882 | 6.435797 |
| hsa-miR-181d | 0.683719 | 1.22001 | down | up | 4.655378 | 4.368486 |
| hsa-miR-27a | 0.691632 | 1.100396 | up | up | 11.82774 | 11.96576 |
| hsa-miR-107 | 0.760746 | 1.069993 | down | up | 10.03742 | 9.939823 |
| hsa-miR-196b | 0.787791 | 2.056219 | up | up | 1.508813 | 2.548807 |
| hsa-miR-200b | 0.804175 | 1.330283 | down | up | 10.12447 | 9.712735 |
| hsa-miR-199a-5p | 0.824088 | 1.083828 | up | up | 10.63369 | 10.74983 |
| hsa-miR-143 | 0.829075 | 1.089806 | down | up | 8.853937 | 8.729867 |
| hsa-miR-429 | 0.856847 | 1.224759 | down | up | 7.325429 | 7.032932 |
| hsa-miR-125a-3p | 0.863100 | 1.096062 | up | up | 6.449531 | 6.58186 |
| hsa-miR-146a | 0.866126 | 1.108128 | down | up | 8.158847 | 8.010722 |
| hsa-miR-23a | 0.873887 | 1.037518 | up | up | 12.4273 | 12.48044 |
| hsa-miR-16 | 0.874665 | 1.034553 | down | up | 11.48334 | 11.43433 |
| hsa-miR-20a | 0.887991 | 1.046063 | down | up | 9.144461 | 9.079493 |
| hsa-miR-181c | 0.907418 | 1.059798 | up | up | 5.025995 | 5.109785 |
| hsa-miR-222 | 0.911535 | 1.06031 | down | up | 7.131722 | 7.047236 |
| hsa-miR-24 | 0.912683 | 1.036835 | up | up | 11.71964 | 11.77182 |
| hsa-miR-23b | 0.936822 | 1.023272 | up | up | 11.14156 | 11.17475 |
| hsa-miR-31 | 0.941169 | 1.230366 | up | up | 3.874156 | 4.173243 |
| hsa-miR-103 | 0.964565 | 1.011592 | down | up | 10.36751 | 10.35088 |
| hsa-miR-221 | 0.969799 | 1.015645 | up | up | 7.43665 | 7.459047 |
| hsa-miR-210 | 0.977395 | 1.042275 | up | up | 6.265573 | 6.325307 |
| hsa-miR-200c | 0.994024 | 1.009893 | down | up | 9.257704 | 9.2435 |
